# Paediatric rheumatology services through the parents’ eyes: a pilot study to develop a disease specific questionnaire to monitor deficiencies of care

**DOI:** 10.1186/1546-0096-9-S1-P124

**Published:** 2011-09-14

**Authors:** A Thon, C Mokroß, F Dressler, K Stahl, W Günther, G Ullrich, G Steinkamp

**Affiliations:** 1 Department of Paediatrics, Hannover Medical School, Germany; 2 Children’s hospital, Oldenburg, Germany; 3 Picker Institute Germany; 4 Clinical Research, Schwerin, Germany

## Background

Considering the patient’s experience is important for developing the best possible chronic illness care. Generic patient surveys do not cover specific aspects of care for the chronically ill, such as the multiprofessional approach, or are restricted to either inpatient or outpatient care.

## Aim

We aimed to develop a disease specific questionnaire for parents of minors with chronic rheumatic diseases.

## Methods

Expert interviews and focus group discussions were carried out in 2 centres to highlight on important dimensions of a new instrument. Audio tapes were used to extract relevant themes. Resulting items were combined with established items of PICKER surveys and so formed the pilot questionnaire. The latter was mailed to all families attending one of the centres during the last year. Responses were analyzed to establish a modified final version.

## Results

Based on focus group discussions new items were identified which were not part of general Picker questionnaires on patient satisfaction, e.g. multi-professional staff, accessibility and support by the center. The pilot questionnaires were mailed to 376 participants, with response rates of 46 %. Only 7 subjects provided suggestions to improve the questionnaires. Although respondents’ satisfaction with chronic illness care was high in general, the reporting style format of items revealed particular aspects of care that should be improved, e.g. insufficient information on side-effects of drugs.

## Conclusion

We have developed a specific questionnaire for chronic rheumatic diseases to investigate parents’ experiences and satisfaction with care. This can be used for quality improvement measures and benchmarking.

